# Elements of Histology

**Published:** 1889-06

**Authors:** 


*Elements of Histology.* By E. KLEIN, M.D., F.R.S. New and Enlarged Edition. Pp. 368. London: CASSELL & Co. 1889.

This is a really admirable specimen of a students' text-book. Written in concise and clear language, arranged in simple and scientific sequences, and abounding in illustrations of the very highest excellence, it is undoubtedly, bulk for bulk, the best work on the subject in the English language.

Some ten reproductions of histological micro-photographs by Mr. Andrew Pringle are given. We fear that the author in speaking of these as "the only really good histological micro-photographs that have as yet been published in any text-book" has been influenced by kindliness of feeling rather than by critical insight. These micro-photographs are certainly the only illustrations in the work that are not really excellent, and they are inferior to others which we have seen in text-books, notably some which have in several works appeared as copied from photographs taken at the United States Army Medical Museum.

